# Author Correction: Filling the gap between topological insulator nanomaterials and triboelectric nanogenerators

**DOI:** 10.1038/s41467-022-29262-z

**Published:** 2022-03-17

**Authors:** Mengjiao Li, Hong-Wei Lu, Shu-Wei Wang, Rei-Ping Li, Jiann-Yeu Chen, Wen-Shuo Chuang, Feng-Shou Yang, Yen-Fu Lin, Chih-Yen Chen, Ying-Chih Lai

**Affiliations:** 1 Department of Materials Science and Engineering, National Chung Hsing University, Taichung, 40227 Taiwan; 2 Department of Physics, National Chung Hsing University, Taichung, 40227 Taiwan; 3 Ming Hsieh Department of Electrical and Computer Engineering, University of Southern California, Los Angeles, CA 90089 USA; 4 Francis Bitter Magnet Lab, Massachusetts Institute of Technology, Cambridge, MA 02139 USA; 5 Department of Materials and Optoelectronic Science, National Sun Yat-Sen University, Kaohsiung, 80424 Taiwan; 6 i-Center for Advanced Science and Technology, National Chung Hsing University, Taichung, 40227 Taiwan; 7 Innovation and Development Center of Sustainable Agriculture, National Chung Hsing University, Taichung, 40227 Taiwan

**Keywords:** Devices for energy harvesting, Electronic devices

Correction to: *Nature Communications* 10.1038/s41467-022-28575-3, published online 17 February 2022.

The original version of this Article contained an error in Fig. 4e, in which the Y-axis was labelled incorrectly as ‘Power density (mW cm^‒2^)’. The correct version of the Y-axis label in Fig. 4e is ‘Power density (mW m^‒2^)’. This has been corrected in both the PDF and HTML versions of the Article.

