# Supplementary material for: Filling the gap between topological insulator nanomaterials and triboelectric nanogenerators
Source: Nat Commun. 2022 Feb 17;13:938. doi: 10.1038/s41467-022-28575-3 (PMC8854595; doi:10.1038/s41467-022-28575-3)
Supplement: Supplementary file 1 — Supplementary Information [file 41467_2022_28575_MOESM1_ESM.pdf]

Supplementary Information

Filling the gap between topological insulator nanomaterials and triboelectric nanogenerators

Mengjiao Li[1,2,3‡], Hong-Wei Lu[1‡], Shu-Wei Wang[1,4], Rei-Ping Li[5], Jiann-Yeu Chen[6,7], Wen-Shuo Chuang[5], Feng-Shou Yang[2], Yen-Fu Lin[2,6*], Chih-Yen Chen[5*], and Ying-Chih Lai[1,6,7*]

Dr. M. J. Li, H.-W. Lu, Dr. S.-W. Wang, Prof. Y.-C. Lai
1 Department of Materials Science and Engineering,
National Chung Hsing University, Taichung, 40227, Taiwan
E-mail: yclai@nchu.edu.tw

Dr. M. J. Li, Dr. F.-S. Yang, Prof. Y.-F. Lin
2 Department of Physics,
National Chung Hsing University, Taichung, 40227, Taiwan
E-mail: yenfulin@nchu.edu.tw

Dr. M. J. Li
3 Ming Hsieh Department of Electrical and Computer Engineering,
University of Southern California, Los Angeles CA 90089, USA

Dr. S.-W. Wang
4 Francis Bitter Magnet Lab, Massachusetts Institute of Technology, Cambridge, Massachusetts 02139, USA

R.-P. Li, W.-S. Chuang, Prof. C.-Y. Chen
5 Department of Materials and Optoelectronic Science, National Sun Yat-Sen University, Kaohsiung, 804, Taiwan
E-mail: cychen@mail.nsysu.edu.tw

Dr. J.-Y. Chen, Prof. Y.-F. Lin, Prof. Y.-C. Lai
6 i-Center for Advanced Science and Technology, National Chung Hsing University, Taichung 40227, Taiwan

Dr. J.-Y. Chen, Prof. Y.-C. Lai
7 Innovation and Development Center of Sustainable Agriculture, National Chung Hsing University, Taichung 40227, Taiwan

[‡] Mengjiao Li and Hong-Wei Lu contributed equally to this work.

Supplementary Figure 1

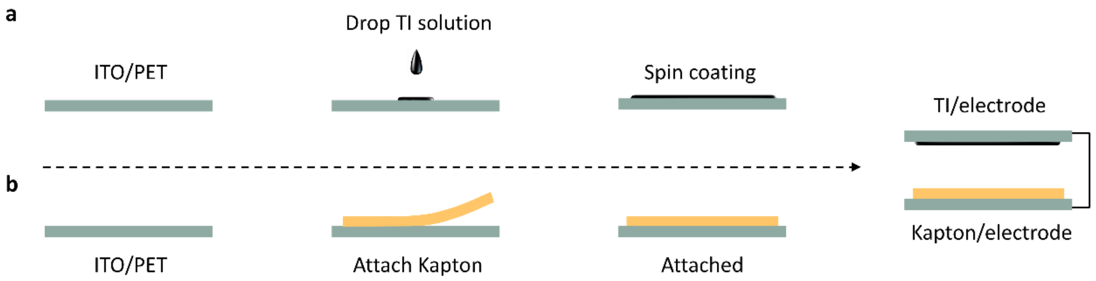

Supplementary Figure 1 | Fabrication process of the TI-TENG using Bi_2Te_3 NPs. Schematic illustration of the fabrication of TI-TENG, which was composed of indium tin oxide (ITO), polyethylene terephthalate (PET), and Bi_2Te_3-NP film, serving as the electrode, substrate, and TI triboelectric layer, respectively. For fabricating the top TI-based triboelectric part (a), the as-synthesized Bi_2Te_3 NPs were firstly dispersed into isopropanol with a suitable concentration to form a homogeneous Bi_2Te_3-NP colloid. A certain dosage of Bi_2Te_3-NP colloid was then uniformly coated on the surface of the PET substrate, which is single-side conductive with ITO film on the bottom. Note that Kapton tape was attached to the substrate all around to mold the Bi_2Te_3-NP film. For fabricating the bottom counterpart (b, taking Kapton as an example), a Kapton film was attached on the top of the PET substrate and ITO also acts as the bottom electrode. Two pieces of triboelectric parts were connected via conductive tape for further measurements.

Supplementary Figure 2

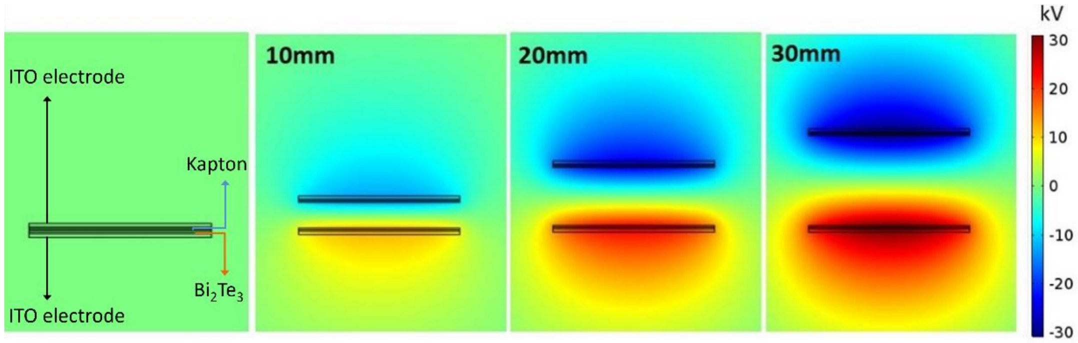

Supplementary Figure 2 | Finite element simulation results. The computed voltage difference between two triboelectric layers of TI film and Kapton via finite element simulation. Note that the simulated spacing between two triboelectric layers was set as 10, 20, and 30 mm, respectively.

Supplementary Figure 3

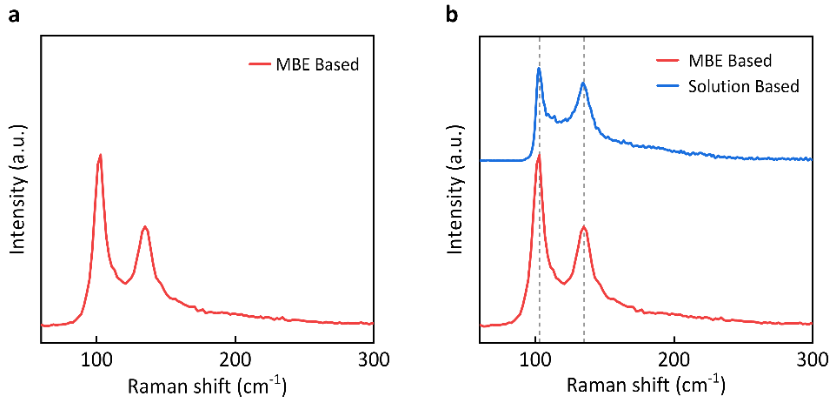

Supplementary Figure 3 | Results of the Raman characterization. Raman spectra of the (a) solution-based Bi_2Te_3 film and (b) MBE-based Bi_2Te_3 film, which indicates its high crystallinity of MBE-grown method. Source data are provided as a Source Data file.

Supplementary Figure 4

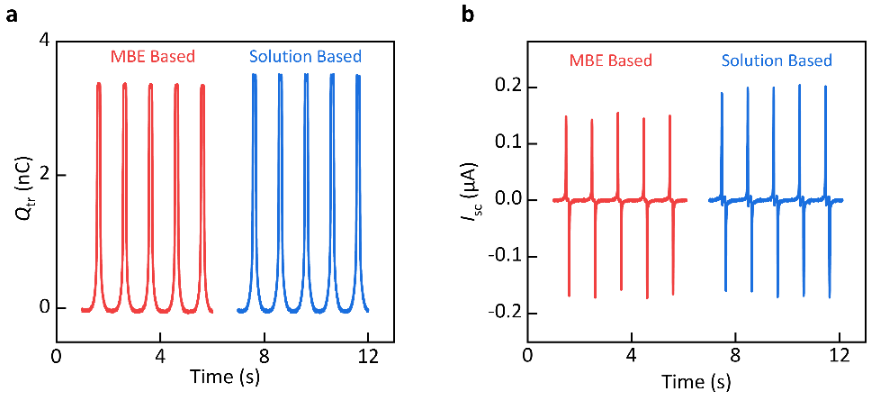

Supplementary Figure 4 | Electric performance of the TI-TENGs with different synthetic methods. Output electrical properties of the MBE-based TI-TENG, including (a) transferred charges and (b) current signal. Note that the film thickness and area of the MBE-grown Bi_2Te_3 are 5 nm and 0.5 cm^2, which are identical with that of the solution-based TI-TENG for a fair comparison. Source data are provided as a Source Data file.

Supplementary Figure 5

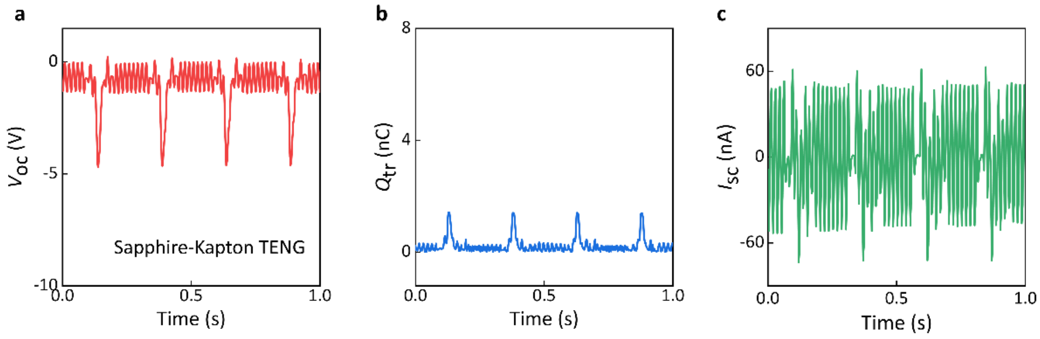

Supplementary Figure 5 | The Controlled experiment of the sapphire-Kapton-based TENG. (a) The measured voltage signal, (b) transferred charges, and (c) measured current signal of the sapphire-Kapton-based TENG, indicating the negligible contributions from the sapphire substrate to the performance of the MBE-based TI-TENG. Source data are provided as a Source Data file.

Supplementary Figure 6

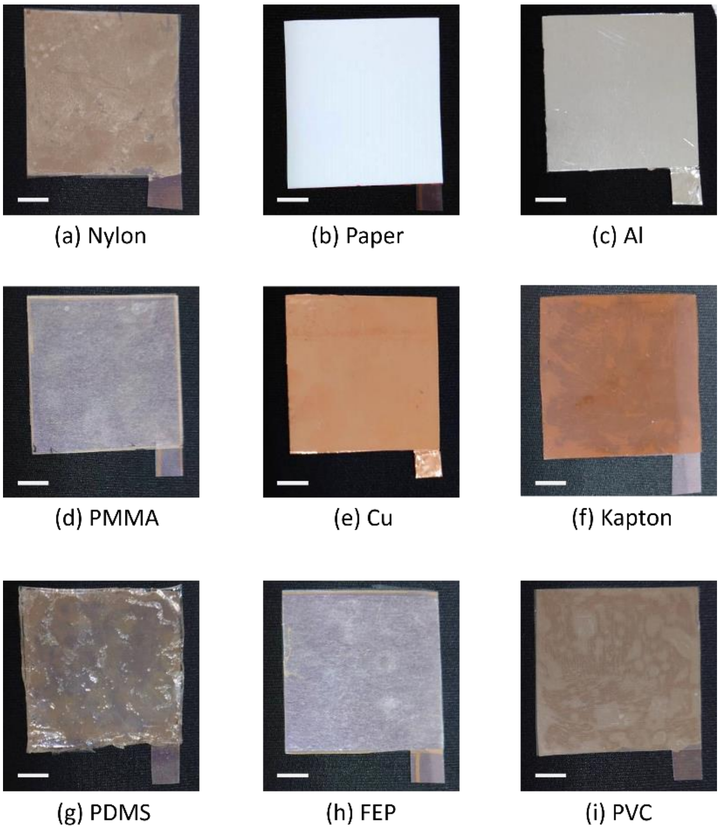

Supplementary Figure 6 | Images of various triboelectric counterparts. Images of the fabricated triboelectric counterparts with various triboelectric media in the current triboelectric series, including (a) nylon, (b) paper, (c) aluminum (Al), (d) polymethyl methacrylate (PMMA), (e) copper (Cu), (f) Kapton, (g) polydimethylsiloxane (PDMS), (h) fluorinated ethylene propylene (FEP), and (i) polyvinyl chloride (PVC), respectively. Scale bar: 1 cm.

Supplementary Figure 7

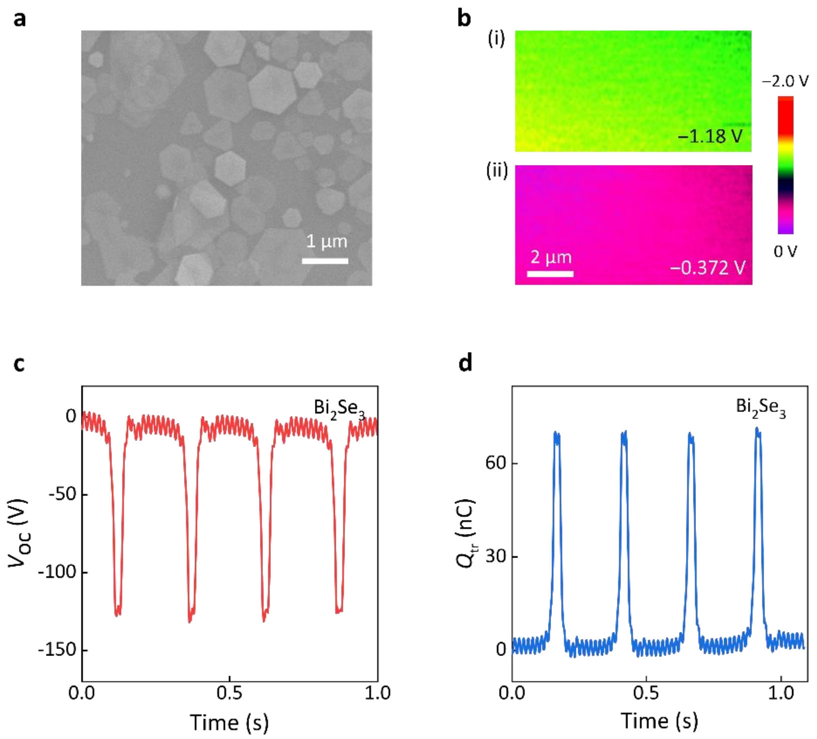

Supplementary Figure 7 The investigation of the Bi_2Se_3 and Bi_2Se_3-NP TI-TENG. (a) SEM image of the hexagonal Bi_2Se_3 nanoplates (Bi_2Se_3 NPs). (b) Surface potential variation of Kapton film (i) before and (ii) after contact with Bi_2Se_3-NP film. (c) and (d) show the measured output voltage and transferred charges of the Bi_2Se_3-NP TI-TENG with Kapton as the counter triboelectric layer. Source data are provided as a Source Data file.

Supplementary Figure 8

Year	Structure of TENGs	Reference
2017	Al/Kapton/MoS_2-Al/PET	Ref. 1
2017	Al/Kapton/rGO-Al	Ref. 2
2018	Al/Kapton-Al-Kapton/Al	Ref. 3
2019	PET/Cu/Kapton-MOF/PET/ITO (Metal-Organic Framework)	Ref. 4
2020	Al/Kapton-MOF/Al	Ref. 5
2020	Kapton-based ternary electrification layered TENG	Ref. 6
2020	CNT/Mxene/silicone rubber-Kapton	Ref. 7
2021	Kapton-Nylon-Copper based alternating mode	Ref. 8
2021	Electrode/Kapton-PET/Electrode rotational TENG	Ref. 9

Supplementary Figure 8 | The collection of the Kapton-based TENGs. It indicates that Kapton is a frequently-used triboelectric material[1-9].

Supplementary Figure 9

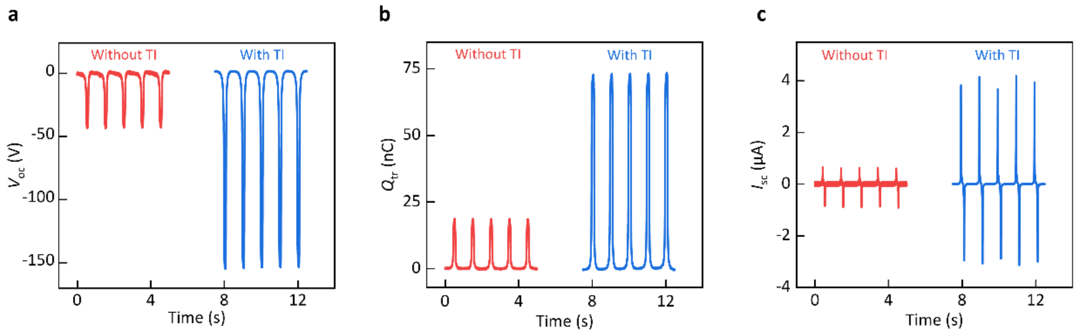

Supplementary Figure 9 | Performance comparison of the TENGs with/without TI films. Comparison plots of the electrical performance (output voltage (a), transferred charges (b), and output current (c)) between different TENGs with and without TI film, which highlight the dominant role of TI films. Source data are provided as a Source Data file.

Supplementary Figure 10

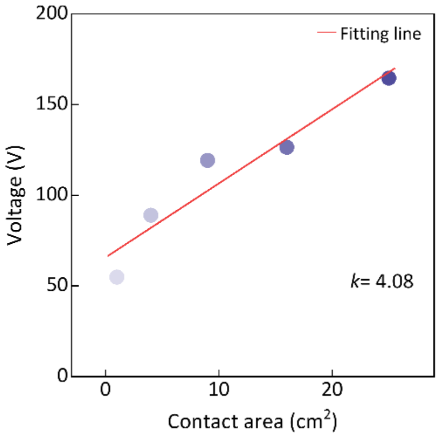

Supplementary Figure 10 | Fitting result of the output voltage versus contact area. The output voltage of the Bi_2Te_3-NP TENG varies depending on the contact area from 1 to 25 cm^2. The fitting line (solid red line) indicates the linear relationship between the voltage signal and the device area. Source data are provided as a Source Data file.

Supplementary Figure 11

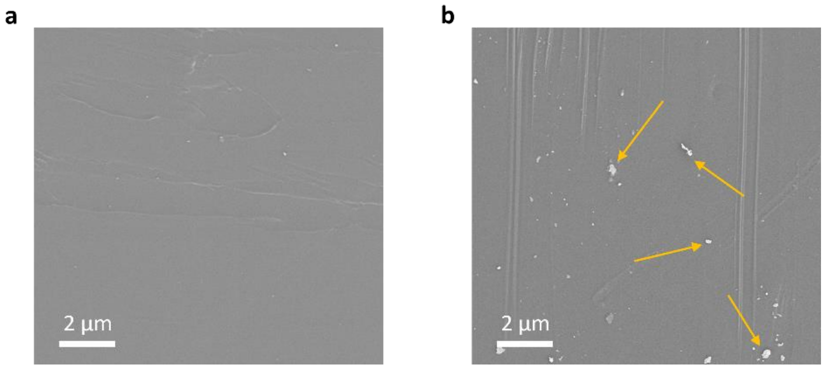

Supplementary Figure 11 | The morphology comparison of the Kapton triboelectric layer after prolonged operation. SEM images of the Kapton film (a) before and (b) after 20000 cycles. The arrows in (b) indicate the few impurities such as Bi_2Te_3 NP on the surface of Kapton after prolonged operation, which lead to negligible impacts on the electrical performance of the TI-TENGs.

Supplementary Figure 12

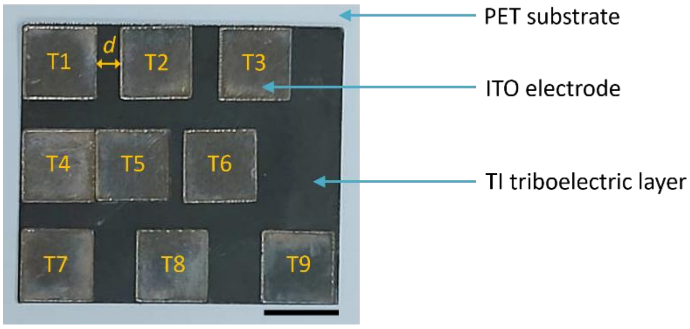

Supplementary Figure 12 | Image of the designed TI-TENG sensor array with different spacings. Image of the bottom side of the designed TI-TENG sensor array, which includes 9 ITO electrodes (1×1 cm^2) with different spacings (d) ranging from 0 to 7 mm. Scale bar: 1 cm.

Supplementary Figure 13

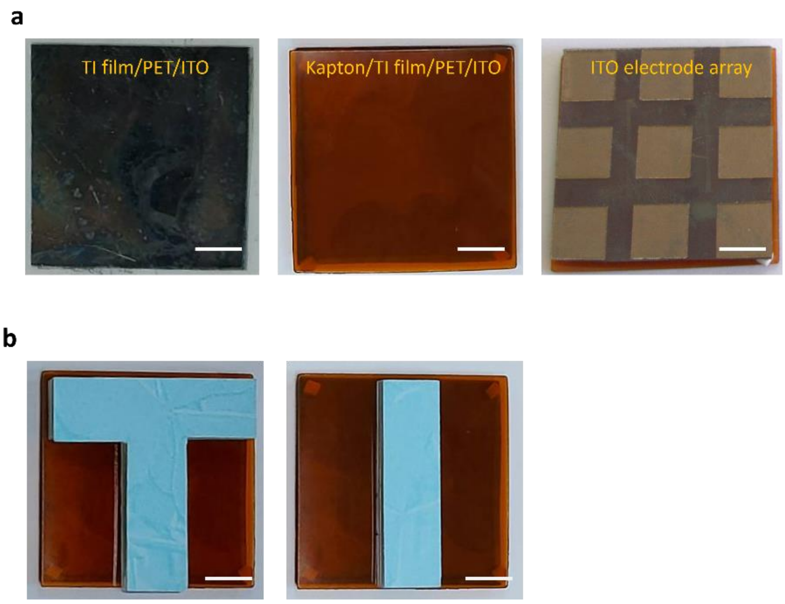

Supplementary Figure 13 | Images of the TI-TENG sensor array with identical spacing. (a) Images of 9 ITO electrodes on the bottom side of the designed TI-TENG sensor array with identical spacing (3 mm). (b) Monitored objects on the TI-TENG sensors with the shapes of letters “T“ and “I“. Scale bar: 1 cm.

Supplementary References

1. C. X. Wu et al., Enhanced Triboelectric Nanogenerators based on MoS_2 Monolayer Nanocomposites Acting as Electron-Acceptor Layers. *ACS Nano* 2017, **11,** 8356.
2. C. X. Wu, T. W. Kim, H. Y. Choi, Reduced graphene-oxide acting as electron-trapping sites in the friction layer for giant triboelectric enhancement. *Nano Energy* 2017, **32,** 542.
3. X. Meng et al., Triboelectric nanogenerator as a highly sensitive self-powered sensors for driver behavior monitoring. *Nano Energy* 2018, **5,** 721.
4. G. Khandelwal, A. Chandrasekhar, N. P. M. J. Raj, S.-J. Kim, Metal-Organic Framework: A novel material for Triboelectric nanogenerator-based Self-powered Sensors and Systems. *Adv. Energy Mater*. 2019, 1803581.
5. G. Khandelwal, N. P. M. J. Raj, S.-J. Kim, Zeolitic imidazole framework: Metal-Organic Framework Subfamily members for Triboelectric Nanogenerator. *Adv. Funct. Mater.* 2020, 1910162.
6. W. Deng et al., Ternary Electrification Layered Architecture for high-performance Triboelectric Nanogenerators. *ACS Nano* 2020, **14,** 9050.
7. W.-T. Cao et al., A stretchable highoutput triboelectric nanogenerator improved by Mxene liquid Electrode with high electronegativity. *Adv. Funct. Mater.* 2020, 2004181.
8. C. Shan et al., An inverting TENG to realize the AC mode based on the coupling of triboelectrification and air-breakdown. *Energy Environ. Sci*. 2021, **14,** 5395.
9. A. Ghaderiaram, A. Bazrafshan, K. Firouzi, M. Kolahdouz, A multi-mode R-TENG for self-powered anemometer under IoT network. *Nano Energy* 2021, **87,** 106170.
