# Supplementary material for: Filling the gap between topological insulator nanomaterials and triboelectric nanogenerators
Source: Nat Commun. 2022 Feb 17;13:938. doi: 10.1038/s41467-022-28575-3 (PMC8854595; doi:10.1038/s41467-022-28575-3)
Supplement: Supplementary file 3 — Description of Additional Supplementary Files [file 41467_2022_28575_MOESM3_ESM.pdf]

Description of Additional Supplementary Files

File Name: Supplementary Movie 1
Description: TI-TENG-based power source. Demonstration of lighting up hundreds of LEDs by a TITENG power source

File Name: Supplementary Movie 2
Description: Storage of electricity and charging of a commercial thermohygrometer. Demonstration of electricity storage process on a bridge circuit and driving of portable electronics like thermohygrometer by self-powered TI-TENG.

File Name: Supplementary Movie 3
Description: Storage of electricity and charging of a portable currency detector. Demonstration of electricity storage process on a bridge circuit driving of portable electronics like UV-light detector by self-powered TI-TENG.

File Name: Supplementary Movie 4
Description: Self-powered game controller. Demonstration of an integrated TI-TENG sensory arraybased game controller.

File Name: Supplementary Movie 5
Description: Self-powered wireless smart glasses. Demonstration of real-time signal sensing ability to serve as a self-powered music controller via a TI-TENG sensory array.
